# A protein-protein interaction dictates Borrelial infectivity

**DOI:** 10.1038/s41598-017-03279-7

**Published:** 2017-06-07

**Authors:** Meghna Thakur, Kavita Sharma, Kinlin Chao, Alexis A. Smith, Osnat Herzberg, Utpal Pal

**Affiliations:** 1 Department of Veterinary Medicine, University of Maryland, College Park and Virginia-Maryland Regional College of Veterinary Medicine, College Park, MD USA; 2 Institute for Bioscience and Biotechnology Research, University of Maryland, College Park, Rockville, USA; 3 Department of Chemistry and Biochemistry, University of Maryland, College Park, Maryland USA

## Abstract

Two *Borrelia burgdorferi* interacting proteins, BB0238 and BB0323, play distinct roles in pathogen biology and infectivity although a significance of their interaction remained enigmatic. Here we identified the polypeptide segment essential for BB0238-BB0323 interaction and examined how it supports spirochete infectivity. We show that the interaction region in BB0323 requires amino acid residues 22–200, suggesting that the binding encompasses discontinuous protein segments. In contrast, the interaction region in BB0238 spans only 11 amino acids, residues 120–130. A deletion of these 11 amino acids neither alters the overall secondary structure of the protein, nor affects its stability or oligomerization property, however, it reduces the post-translational stability of the binding partner, BB0323. Mutant *B*. *burgdorferi* isolates producing BB0238 lacking the 11-amino acid interaction region were able to persist in ticks but failed to transmit to mice or to establish infection. These results suggest that BB0238-BB0323 interaction is critical for post-translational stability of BB0323, and that this interaction is important for mammalian infectivity and transmission of *B*. *burgdorferi*. We show that saturation or inhibition of BB0238-BB0323 interaction could be studied in a luciferase assay, which could be amenable for future identification of small molecule inhibitors to combat *B*. *burgdorferi* infection.

## Introduction

Lyme borreliosis, caused by the spirochete bacteria *Borrelia burgdorferi* senso lato, is a common tick-borne illness in many parts of the world^1, 2^. The global prevalence of the disease has been steadily growing, with nearly 25-fold increase in new cases over the last two decades. Lyme disease is severely underreported, as according to a recent estimate by Center of Disease Control and Prevention, the average numbers of yearly cases in the United States alone are likely to be at least 300,000, which is 10-fold higher than the previous estimate. Moreover, new and virulent strains of Lyme disease pathogens have been discovered recently^3, 4^. While majority of patients treated early with antibiotics usually recover, the late-stage patients require longer antibiotic treatment. Notably, a subset of antibiotic-treated patients experience complications collectively referred to as post treatment Lyme disease syndrome (PTLD) or chronic Lyme disease^5, 6^. The etiology of PTLD, its pathogenic mechanism or treatment options remain unknown. Given the clinical challenges associated with management of PTLD cases, development of novel preventive strategies and treatments, including vaccines and antimicrobials are highly warranted.

Protein-protein interactions mediate key biological functions thereby offering a plethora of unexplored potential for next-generation drug targets^7, 8^. Indeed, the bacterial or host-pathogen protein interactomes provide invaluable insights for the identification of novel antimicrobial drug targets, as successfully accomplished in cases of many human pathogens^8–10^. A wealth of data involving host-*B*. *burgdorferi* interaction has also been accumulated over the past decade^11^, although physical and functional attributes of such interactions or how they contribute to spirochete biology and infectivity remain unknown. Insight into these areas will not only facilitate our understanding of key molecular mechanisms that govern microbial biology, infectivity and pathogenesis, but will also provide valuable cues and alternative approaches for development of new drug targets. A previous study also explored a global identification of protein-protein interaction in spirochete outer membrane (OM), which revealed a remarkable number of protein complexes of undefined biological significance^12^. Notably, compare to other bacteria, *B*. *burgdorferi* OM composition and cellular organization feature distinct molecular characteristics^13^. For example, the OM lacks phosphatidylethanolamine and classical lipopolysaccharide and has relatively low abundance of membrane-spanning proteins, including Braun’s lipoprotein or transenvelope spanning protein complexes such as the Tol-Pal system that connects OM to the underlying peptidoglycan (PG) layer surrounding the inner membrane (IM). As the periplamic endoflagella run between the OM and inner membrane (IM), it is still unclear how the OM is organized over the flagella and is physically linked to the PG layer or IM^14^. In addition, *B*. *burgdorferi* cycles between diverse mammalian and arthropod tissue environments, where the pathogen undergoes constant antigenic alterations^15^. It remains to be determined how protein-protein interactions support microbial biology and infectivity through a complex enzootic infection cycle.

The *B*. *burgdorferi* BB0323 protein has recently been identified, and shown to play a key role in stability of the spirochete OM, cell fission and infectivity^16–19^. Apart from the presence of a single lysin motif (LysM) at the C-terminus, BB0323 does not share homology with any known protein. The protein undergoes multi-step proteolytic processing to yield mature N-terminal and C-terminal polypeptides that have indispensable roles in cell fission and mammalian infectivity^17^. More recently, we have discovered that BB0323 interacts with another *B*. *burgdorferi* protein of unknown function, BB0238, both of which are indispensable for spirochete infectivity of the murine hosts^16^. Here we identify the BB0238-BB0323 binding regions and show that the interaction between the two proteins is essential for successful establishment of spirochete infection or transmission. We speculate that blocking this interaction provides a novel strategy to combat Lyme borreliosis.

## Results

### BB0238 interacts with BB0323 via 11-residues spanning amino acids 120–130

**Figure 1:**
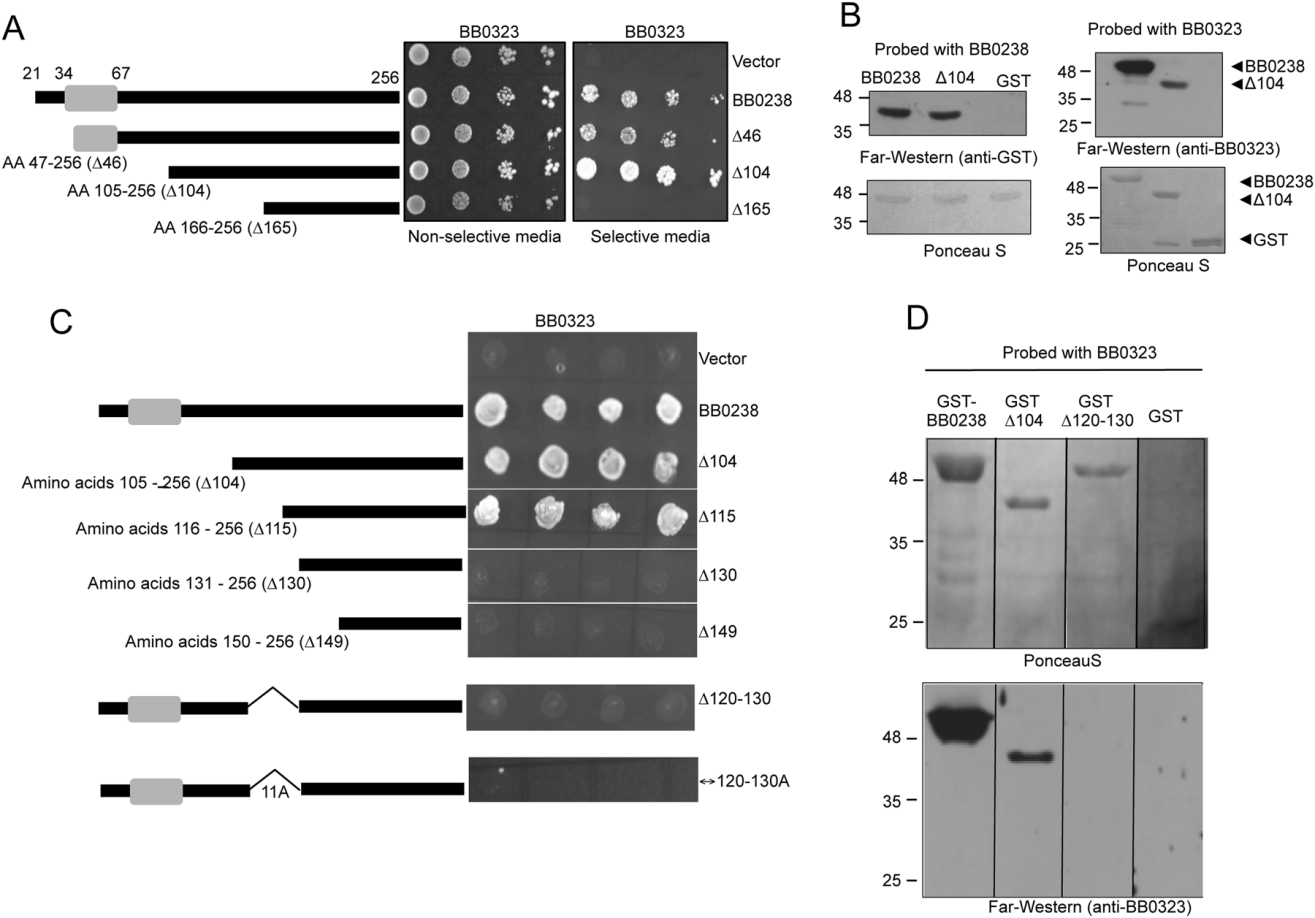
Probing the BB0238-BB0323 interaction. (**A**) Residues 34–67 in BB0238 are not involved in the interaction with BB0323. Yeast two-hybrid assays for assessment of BB0238-BB0323 interaction. The yeast vector pGAD expressing GAL4 activation domain (AD) or various fusions of AD-BB0238 were introduced into yeast along with the vector pGBKT7 expressing the GAL4 DNA binding domain (BD) fused with BB0323. Serially diluted transformants were grown in non-selective (SD-Trp-Leu) or selective (SD–Ade–His–Leu–Trp) media and yeast growth was recorded. (**B**) BB0323 and BB0238 interact in Far-Western assays. Recombinant BB0323 (left panels) or BB0238 (right panels) was subjected to SDS-PAGE and incubated with glutathione S-transferase (GST)-fused BB0238, or His-tagged BB0323 proteins, and binding was examined by appropriate primary and secondary detection antibodies. (**C**) Residues 120–130 of BB0238 mediate the interaction with BB0323. Yeast vector expressing indicated AD-BB0238 fusion proteins were cotransformed with BD-BB0323 and growth was assessed, as detailed in panel A. (**D**) BB0238 missing 11-residue interaction motif failed to bind BB0323 in far-Western assays. Recombinant GST-BB0238 fusion proteins were subjected to SDS-PAGE and incubated with recombinant his-tagged BB0323 and the interaction was monitored as described in panel B. Migration of protein standards is shown to the left in kilodaltons.

We have previously shown that two *B*. *burgdorferi* proteins, BB0323 and BB0238, interact with and stabilize one another, and either partners play indispensable roles in spirochete infectivity^16, 17^. To further characterize the interaction, we sought to identify the binding regions in both proteins. Residues 34–67 are presumed to harbor a known protein-protein interaction motif called tetratricopeptide repeat in BB0238 although our secondary structure predictions using Robetta^20^ and I-tasser^21^ did not support that notion. Nevertheless, as site directed mutagenesis, targeting residues within this range, impaired spirochete infectivity^22^, we tested whether this region plays a role in binding to BB0323. To accomplish this, we produced truncated BB0238 with incremental N-terminal deletions of 47, 104 and 165 residues and assessed whether the truncated proteins interacted with BB0323 using a yeast two-hybrid assay. As shown in Fig. 1A, neither partial nor full deletion of the region (missing the N-terminal 46 residues or 104 residues denoted as Δ46 or Δ104, respectively) impaired the interaction, as evidenced by the growth of yeast transformants on selective media (Fig. 1A). A similar result was also obtained by an independent far-Western assay, as shown in Fig. 1B. Taken together, these studies suggested that residues 34–67, presumed to harbor a TPR motif^22^, do not play a role in interaction with BB0323.

In contrast, the deletion of 165 residues from the N-terminus of BB0238 abolished the interaction with BB0323, indicating that the interaction region is contained within residues 104–165 (Fig. 1A). To further narrow the interaction region, we generated additional serial deletions and showed that residues 116–130 of BB0238 are important for interaction with BB0323 (Fig. 1C). An additional mutant with an internal deletion of 11 amino acids KIEYIAQRERS, designated Δ120–130, was also unable to interact with BB0323. A far-Western assay confirmed that unlike wild type BB0238, the mutant protein lacking 11 amino acids (Δ120–130) was unable to bind BB0323 (Fig. 1D). Moreover, replacement of these 11 amino acids by a stretch of 11 alanine residues also abolished the interaction (Fig. 1C), suggesting that the nature of the amino acid side chain(s) is critical to the BB0238-BB0323 interaction. We next replaced either single or combinations of amino acids within the interaction segment (KIEYIAQRERS) by alanine residues. However, none of the point or combination mutants fully disrupted the interaction, although replacement of the KIEYI sequence significantly impaired the interaction (Supplementary Fig. S1). Since the binding epitope could not be attributed to a single or a few amino acid residue(s), we used the BB0238 protein devoid of 11 residues, hereafter referred to as BB0238^*∆IM*^, for subsequent analysis.

### BB0323 interaction with BB0238 is mediated by non-continuous amino acid segments spanning residues 22–200

**Figure 2:**
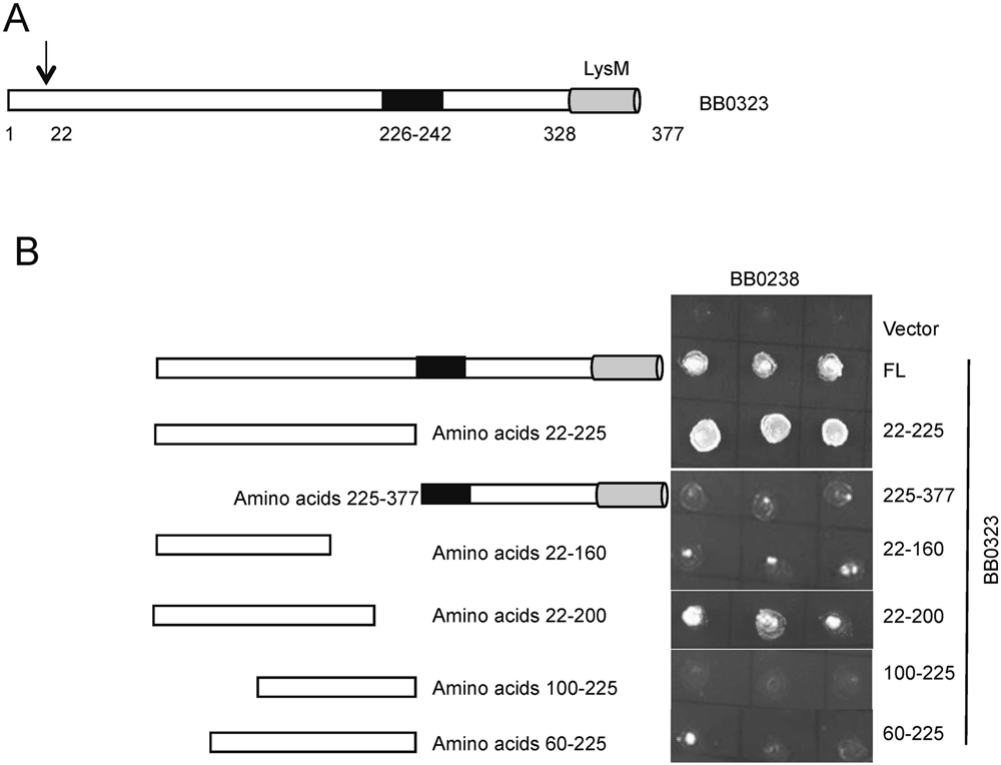
BB0238 interaction region in BB0323 encompasses the 200 N-terminal amino acids. (**A**) Schematic representation of full-length BB0323. Arrow indicates the predicted signal peptide cleavage site. Residues 226–242 indicate the interaction region between the N- and C-terminal BB0323 polypeptides^17^. (**B**) Yeast two-hybrid assays to identify the BB0238-BB0323 interaction region. Truncated BB0323 fusion proteins, as schematically represented (left panels) were expressed in yeast as BD-BB0323 fusion proteins that were cotransformed with AD-BB0238 and yeast growth was monitored as detailed in Fig. 1A.

Next we sought to define the BB0323 region that binds to BB0238 using a yeast two-hybrid assay. In agreement with published data^16^, the N-terminal domain of BB0323 (residues 22–225), not the C-terminal domain (residues 225–377), readily interacted with BB0238 (Fig. 2B). To further narrow down the BB0323 interaction region, we generated various truncations of the protein and studied the binding. The results showed that the minimum region of BB0323 that interacted with BB0238 spanned residues 22–200, and any truncation either form the N- or C-terminus was deleterious for its interaction with BB0238 (Fig. 2B). These data suggest that the BB0323 binding site stretches throughout the N-terminal and center region of the polypeptide chain.

### BB0238 binding site is not important for protein stability, structure integrity, or oligomerization

**Figure 3:**
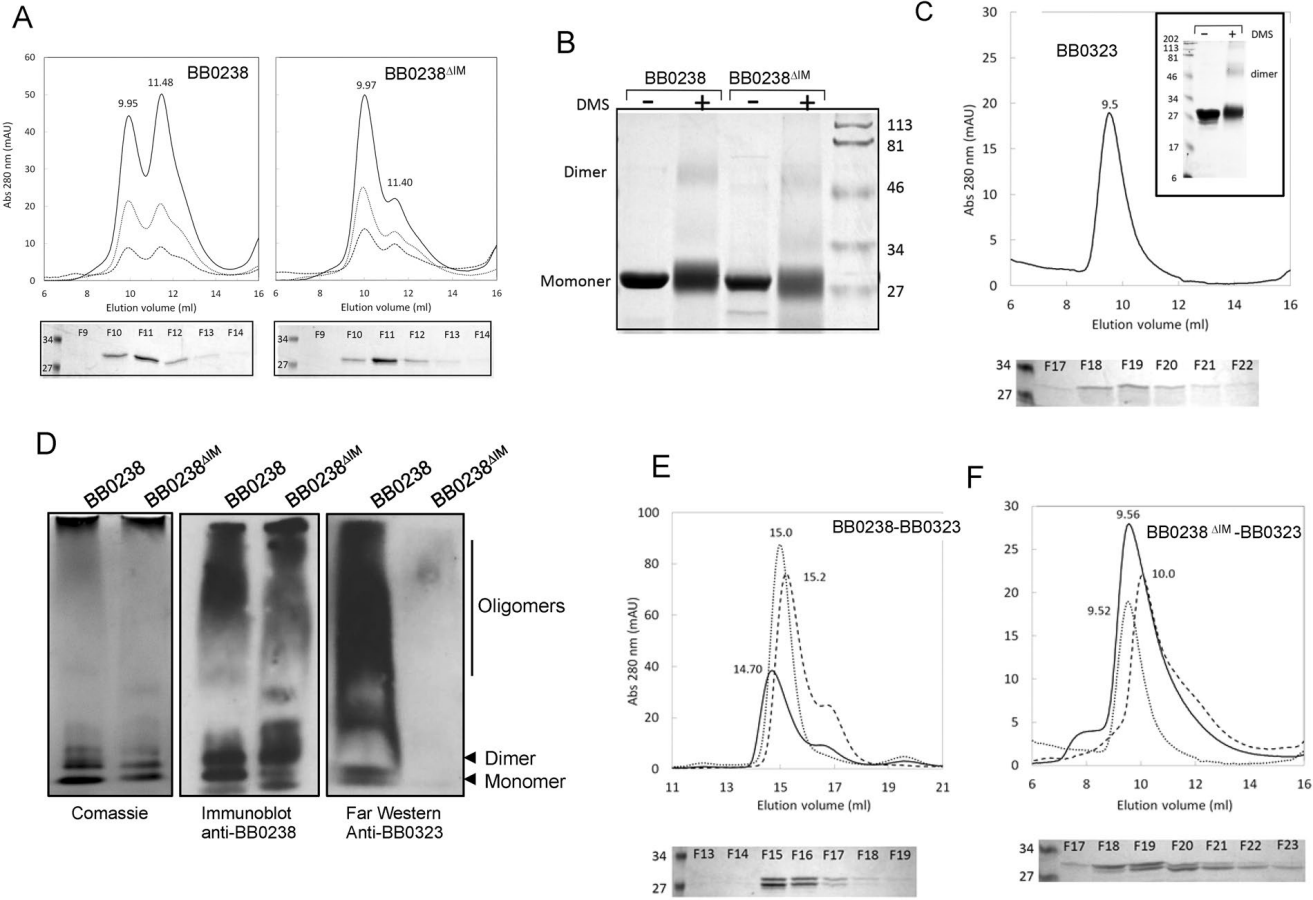
Homo- and hetero-oligomerization of BB0323 and BB0238 in the presence and absence of BB0238 11-residue interaction region. (**A**) Quaternary structure of BB0238 and BB0238^∆IM^ as assessed by analytical size exclusion chromatography. Elution profiles of various concentrations of proteins, 9 μM (dashed line), 18 μM (dotted line) and 44 μM (solid line) BB0238 (left panel) and 10 μM (dashed line), 20 μM (dotted line) and 40 μM (solid line) BB0238^∆IM^ (right panel) from Sephadex 75 column. Lower panels show coomassie-stained denaturing SDS-PAGE of the eluted fractions F9-F14 from the experiments using 44 μM BB0238 or 40 μM BB0238^∆IM^. (**B**) BB0238 Cross-linking studies. Coomassie-stained denaturing SDS-PAGE of ~9 μM BB0238 and BB0238^∆IM^ cross-linked with DMS as described in Materials and Methods. Lanes labeled (−) and (+) correspond to proteins that were treated with 1 M Triethanolamine buffer alone or with DMS, respectively. The right lane shows molecular weight markers. (**C**) Quaternary structure of BB0323 (residues 22–242) as assessed by analytical size exclusion chromatography. Elution profiles of 8 μM BB0323 from Superdex 75 column. Bottom panel showed coomassie blue-stained denaturing SDS-PAGE of BB0323 N2 in 0.5 mL fractions collected. Inset: Coomassie blue-stained denaturing SDS-PAGE of ~19 μM BB0323 crosslinked with DMS as described in Materials and Methods. BB0323 in the negative control (−) and positive (+) lanes were treated with 1 M Triethanolamine buffer alone or with DMS, respectively. (**D**) One dimensional PAGE separation of native BB0238 and BB0238^∆IM^. Gel separations were stained with Comassie Brilliant Blue (left panel), immunoblotted with anti-BB0238 antibody (middle panel) and subjected to far-Western analysis using anti-BB0323 antibody (right panel). (**E**) Elution profiles of 10 μM BB0238- BB0323 complex at ~1:1 molar ratio (solid line), 30 μM BB0323 (dotted line) and 40 μM BB0238 (dashed line) from Superdex 200 column with 1 mL fractions collected. (**F**) Elution profiles of ~9 μM BB0238^*∆IM*^-8 μM BB0323^22-242^complex (solid line), 8 μM BB0323 (dotted line) and 9 μM BB0238^*∆IM*^ (dashed line) from Superdex 75 column with 0.5 mL fractions collected.

Our initial studies using Far-UV circular dichroism (CD) spectroscopy of the wild-type BB0238 and the mutant protein (BB0238^*∆IM*^) suggested that the deletion of 11 residues did not affect the secondary structure of the protein (Supplementary Fig. S2). Analytical size exclusion chromatography next showed that BB0238 exists in solution as a mixture of monomers and dimers. As protein-protein interaction could be dependent on the oligomeric status of either partner, we assessed whether deletion of the 11 residues in BB0238^*∆IM*^ impaired its ability to form dimers by comparing the quaternary structure of the wild type and BB0238^*∆IM*^ proteins. Using the matrix-assisted laser desorption time-of-flight (MALDI-TOF) mass spectrometry, the molecular masses of purified BB0238 and BB0238^*∆IM*^ were determined to be 27,627 ± 23 Da and 26,267 ± 39 Da respectively. The elution profiles of intact BB0238 from Sephadex 75 column showed two approximately equal peaks at 9.93 ± 0.02 mL and 11.45 ± 0.03 mL at a concentration range of 9–44 μM (Fig. 3A, left panel). Based on the protein standard curve and assuming globularly shaped molecules, these peaks are consistent with molecular weights of 40.8 kDa and 22 kDa, respectively, indicating a monomer-dimer equilibrium. Likewise, using a 10–40 μM concentration range, BB0238^*∆IM*^ also eluted as two main peaks at 10.0 ± 0.03 ml and 11.39 ± 0.02 ml with an apparent molecular weight of 40.5 kDa and 22.8 kDa (Fig. 3A, right panel). The eluted fractions were analyzed with denaturing SDS-PAGE to confirm the presence of intact BB0238 and BB0238^*∆IM*^ proteins in the dimer peaks (fractions F10-F11) (Fig. 3A, lower panels). Thus, both BB0238 and BB0238^*∆IM*^ form dimers and the loss of 11 residues in BB0238^*∆IM*^ did not impair or perhaps even enhanced the oligomerization process. The relative monomer and dimer peak heights suggest that the BB0238 dimer may be weaker or more dynamic than that of BB0238^*∆IM*^.

Additional support for the dimerization of both BB0238 and BB0238^*∆IM*^ was obtained by the treatment of the proteins with dimethylsuberimidate (DMS), a reagent that covalently crosslinks oligomeric proteins through interactions with neighboring lysine residues. The products of the crosslinking reaction for both BB0238 and BB0238^*∆IM*^ showed a higher molecular weight species that migrated just above the 46-kDa marker protein on denaturing SDS-PAGE (Fig. 3B).

We next analyzed the quaternary structure of BB0323 by analytical size exclusion chromatography. Our previous studies have shown that the region comprising residues 22–242 of BB0323, denoted BB0323 N2, is able to interact with the cleaved C-terminal domain (residues 243–377) and harbors the unidentified cleavage site for genesis of mature polypeptides (Kariu *et al*.^17^). Additionally this region retains the ability of BB0323 to bind BB0238. The analytical size exclusion chromatography of the BB0323 N2 domain, performed with Superdex 75, showed a single peak eluted at ~9.5 ml (Fig. 3C). Based on the calibration curve, this peak corresponds to an apparent molecular weight of 48.5 kDa, indicating a stable BB0323 dimer. The treatment of BB0323 with dimethylsuberimidate (DMS) produced a crosslinked dimer species that migrated just above 46-kDa marker on denaturing SDS-PAGE (inset Fig. 3C), in agreement with the size exclusion chromatography results.

In addition to size exclusion chromatography and crosslinking experiments, oligomeric forms of BB0238 and BB0238^*∆IM*^ were also visible on native PAGE, and were interpreted as corresponding to the monomeric and dimeric forms (Fig. 3D left panel). Interestingly, the immunoblot and far-Western using anti-BB0238 and anti-BB0323, respectively (Fig. 3D middle and right panels) revealed aggregation under the native PAGE conditions. Nevertheless, BB0238 showed binding to BB0323 regardless of the oligomeric form, whereas binding was abolished in the mutant despite the presence of oligomeric species on the native PAGE. The far-Western also shows that even the monomeric BB0238 binds BB0323, consistent with a stoichiometric ratio of 1:1.

Analytical size exclusion chromatography supports the association of a 1:1 BB0238:BB0323 complex and the absence of BB0238^*∆IM*^:BB0323 complex (Fig. 3E and F). When BB0323 and BB0238 were mixed at stoichiometric ratio (~10 μM), the elution profile from a Superdex 200 column showed that the peak shifted to 14.7 mL compared with the reference elution peaks of BB0323 (at 15 mL) and BB0238 (at 15.20 and 16.86 mL). A 2:2 hetero-tetramer of ~110 kDa would be expected to elute significantly earlier. In contrast, the elution profile of the 1:1 mixture of BB0323 and BB0238^*∆IM*^ showed no significant shift in the peak compared with the elution peaks of the two proteins alone, confirming the lack of protein-protein interaction (Fig. 3F).

**Figure 4:**
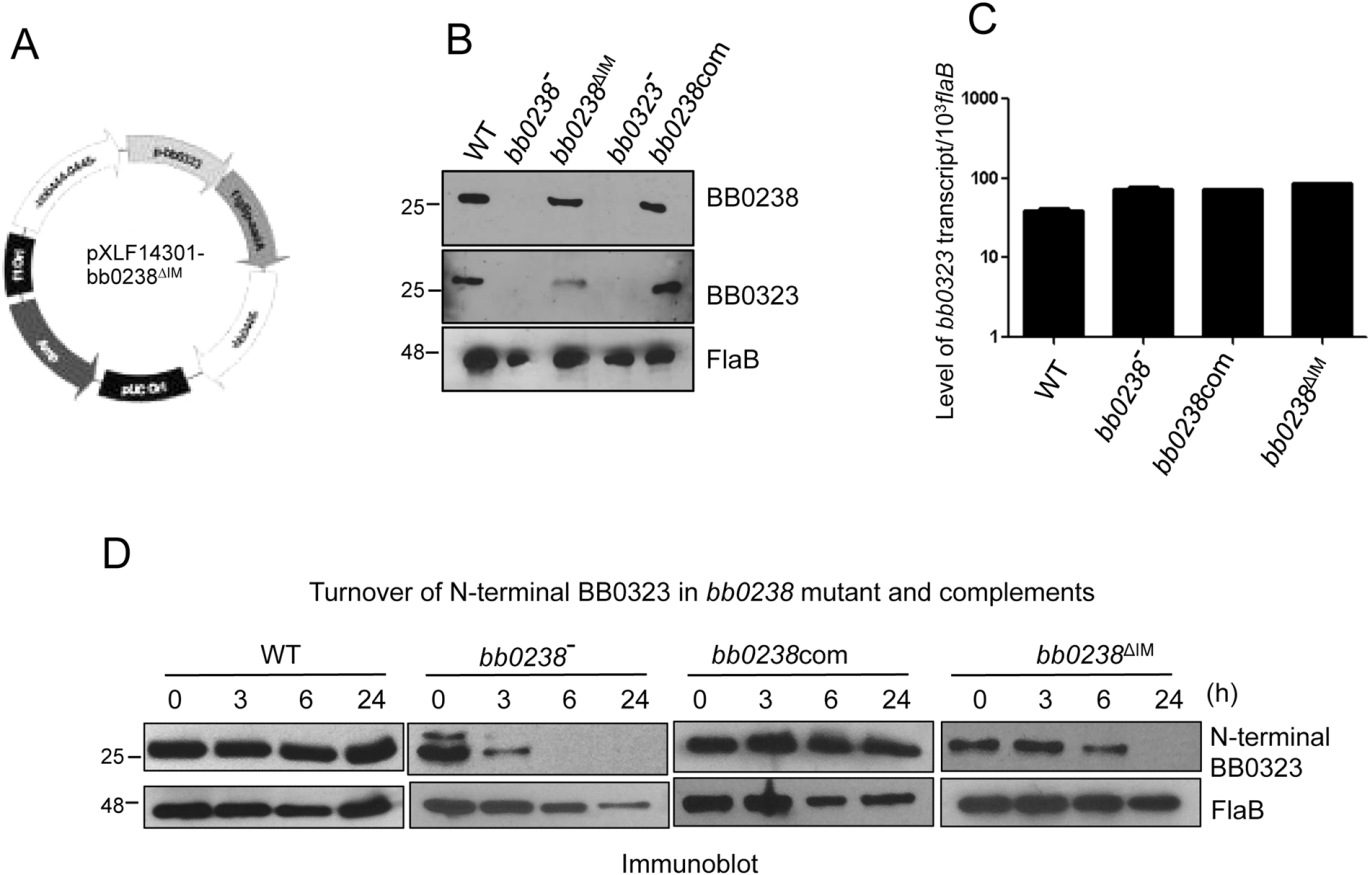
The interaction with intact BB0238 is required for the stability of BB0323 in *B*. *burgdorferi*. (**A**) Diagram of vector construct (pXLF14301- bb0238^∆IM^) for chromosomal insertion of *bb0238* ^*∆IM*^. The construct was created by deletion mutagenesis of the previously used *bb0238* complement construct^16^. (**B**) Production of BB0238 and BB0323 proteins by the *bb0238* ^*∆IM*^ complemented *B*. *burgdorferi*. Lysates of wild type (WT), *bb0238* mutant (bb0238^−^), *bb0238* complement (bb0238com), *bb0238* ^*∆IM*^ complement (bb0238^∆IM^) and *bb0323* mutant (bb0323^−^) *B*. *burgdorferi* were separated by SDS-PAGE, transferred on nitrocellulose membrane and blotted with antiserum against BB0238 (upper panel), BB0323 (middle panel) and FlaB (lower panel). (**C**) Expression of BB0238^∆IM^ did not alter the transcript level of *bb0323*. The level of *bb0323* transcripts relative to *flaB* in WT, *bb0238* mutant, *bb0238* complement and *bb0238* ^*∆IM*^ complement was measured by qRT-PCR. (**D**) Posttranslational stability of BB0323 in WT, *bb0238* mutant, *bb0238* and *bb0238* ^*∆IM*^ complemented *B*. *burgdorferi*. Protein synthesis was inhibited by the addition of 100 µg/ml spectinomycin to growing cultures of spirochetes, and levels of N-terminal domain of BB0323 were determined by immunoblot analysis. At indicated time points, spirochetes were collected, lysates were separated by SDS-PAGE and immunoblotted using anti-BB0323 and anti-FlaB (loading control) antisera. Migration of protein standards is shown to the left in kilodaltons.

We next examined the relevance of the interaction region between BB0238 and BB0323, as identified by the *in vitro* studies, to BB0323 stability in *B*. *burgdorferi*. Our previous studies have established that deletion of *bb0238* from the genome of *B*. *burgdorferi* destabilized the interaction partner BB0323, and stability is recovered in a genetically-complemented isolate generated by inserting the wild type copy of the gene^16^. To understand the importance of the protein-protein interaction for the stability of BB0323, we complemented the *bb0238* deletion mutant with *bb0238* ^*∆IM*^ (Fig. 4A). First we confirmed that the deletion of the 11-residue interaction segment did not abolish the expression of the mutated protein in *B*. *burgdorferi*. As shown in Fig. 4B (upper panel), the expression level of the mutant BB0238^∆IM^ was similar to that of the native protein either in wild type or *bb0238* complemented isolates. However, the amount of the interacting partner protein, BB0323, was significantly reduced (Fig 4B, middle panel). In contrast, the *bb0323* transcript levels remained unaffected (Fig. 4C), indicating that the interaction with BB0238 contributes to the posttranslational stability of BB0323.

The reduced BB0323 stability in the presence of BB0238^*∆IM*^ was further corroborated by investigating the protein turnover in *bb0238* ^*∆IM*^ *B*. *burgdorferi*. Spirochetes were treated with spectinomycin to inhibit protein synthesis and the amount of BB0323 was monitored at different time point following treatment. As shown in Fig. 4D, BB0323 cell content remained stable up to 24 hour following inhibition of protein synthesis in the wild type or *bb0238* complemented spirochetes, however, the protein was dramatically degraded in cells either lacking intact *bb0238* or in cells engineered to express the *bb0238* ^*∆IM*^ gene. Therefore, the protein-protein interaction is critical for preventing the cellular degradation of BB0323.

### Impaired BB0238-BB0323 interaction diminishes spirochete ability to persist in mice or their transmission from infected ticks to naïve hosts

**Figure 5:**
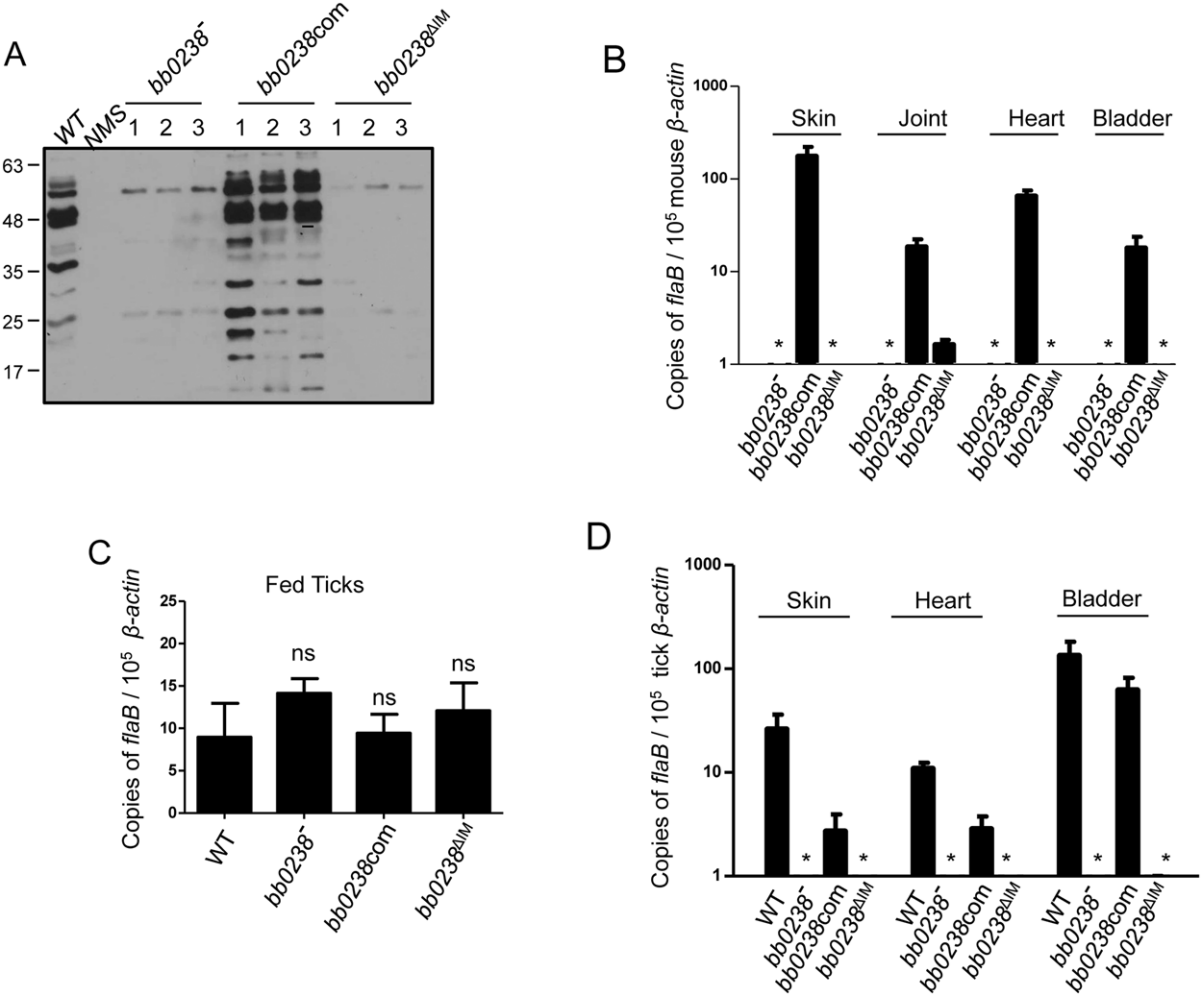
Deletion of the 11-residues of BB0238 that interact with BB0323 impaired spirochete ability to persist in mice or their transmission from infected ticks to naïve hosts. (**A**) Mice (3 animals/group) were infected with *bb0238* mutant (bb0238^−^), *bb0238* complement (bb0238com) and *bb0238* ^*∆IM*^ complement (bb0238^∆IM^) spirochetes. Sera collected 2 weeks post inoculation was used to screen for immunoblot of *B*. *burgdorferi* cell lysate. NMS denotes normal mouse sera, used as a control. (**B**) Spirochete burdens in infected mice were assessed by qRT-PCR, by measuring copies of *B*. *burgdorferi flab* gene normalized to mouse *β-actin* levels in skin, joint, heart and bladder. Error bars represent mean standard errors from three independent experiments. (**C**) Ticks were microinjected with equal numbers of wildtype (WT), *bb0238* mutant, *bb0238* complement, or *bb0238* ^*∆IM*^ complemented *B*. *burgdorferi* and allowed to engorge on mice (3 animals per group). Spirochete burdens in fully engorged ticks (**C**) and different mice tissues (**D**) were analyzed with qRT-PCR after 2 weeks of infection by measuring copies of *flaB* normalized against tick or mouse *β-actin* respectively. Asterisks indicate undetectable levels of *bb0238* mutant and *bb0238* ^*∆IM*^ complement. Statistical significance of differences were analyzed using GraphPad Prism v5. One- or two-tailed Student’s t tests were used to compare the mean values, and p < 0.05 was considered statistically significant.

We next sought to determine whether BB0238-BB0323 interaction is important for borrelial virulence and persistence through tick-mouse infection cycle. We assessed whether *B*. *burgdorferi* lacking the 11-residue BB0323 interaction region in BB0238 could establish infection in murine hosts and transmit from ticks to mice. Groups of rodents (three animals/group) were infected either with spirochetes bearing the *bb0238* deletion mutant that is unable to produce BB0238, *bb0238* ^*∆IM*^ mutant that produces BB0238^*∆IM*^, or the *bb0238* complemented spirochetes that express wild-type BB0238. As shown in Fig. 5A, while mice infected with *bb0238* complemented spirochetes displayed strong immune responses suggesting active infection, the *bb0238* deletion mutant or *bb0238* ^*∆IM*^ isolate failed to evoke detectable antibody responses indicating that the 11-residue deletion in BB0238^*∆IM*^ renders spirochetes non-infectious in mice. In agreement with the serological response, the qRT-PCR analyses of pathogen level in murine hosts at two weeks after needle inoculation indicated that unlike *bb0238* complemented controls, *bb0238* deletion mutants or *bb0238* ^*∆IM*^ isolates remained undetectable in murine tissues (Fig. 5B). In addition, the culture analysis, assessed at weekly intervals until four weeks, showed that both *bb0238* mutants and *bb0238* ^*∆IM*^ remained undetectable, although both wild type and *bb0238* complemented spirochetes persisted in skin and spleen samples collected form 5 mice (4 of 5). To rule out anomalous effects of genetic manipulation of *B*. *burgdorferi*, we tested multiple clones from independent transformations for loss of infectivity of various *bb0238* ^*∆IM*^ isolates. All tested mutant clones produced BB0238 lacking the BB0323 interaction region but were unable to infect the host (Supplementary Fig. S3).

To assess whether deletion of the interaction region in *bb0238* ^*∆IM*^ isolates affect their ability to transmit from infected ticks to naïve hosts, groups of spirochetes were directly introduced in the vector via microinjection, and ticks were allowed to parasitize naïve C3H mice (three animals/group). Fully repleted ticks were collected and pathogen burden was monitored using qRT-PCR. As shown in Fig. 5C, all the groups of *B*. *burgdorferi*, including the *bb0238* ^*∆IM*^ isolates exhibited similar persistence in ticks indicating that the BB0238-BB0323 interaction does not play a role in spirochete survival in the arthropod vector. Fourteen days after tick feeding, analysis of *B*. *burgdorferi* infection in various host tissues, including skin, heart and bladder showed that unlike the control spirochetes carrying a wild type copy of *bb0238*, either the *bb0238* deletion mutants or the *bb0238* ^*∆IM*^ isolates failed to establish infection in the hosts (Fig. 5D). These data establish that the BB0238-BB0323 association, mediated by residues 120–130 of BB0238, is necessary for transmission of the pathogens to the murine host via tick feeding.

**Figure 6:**
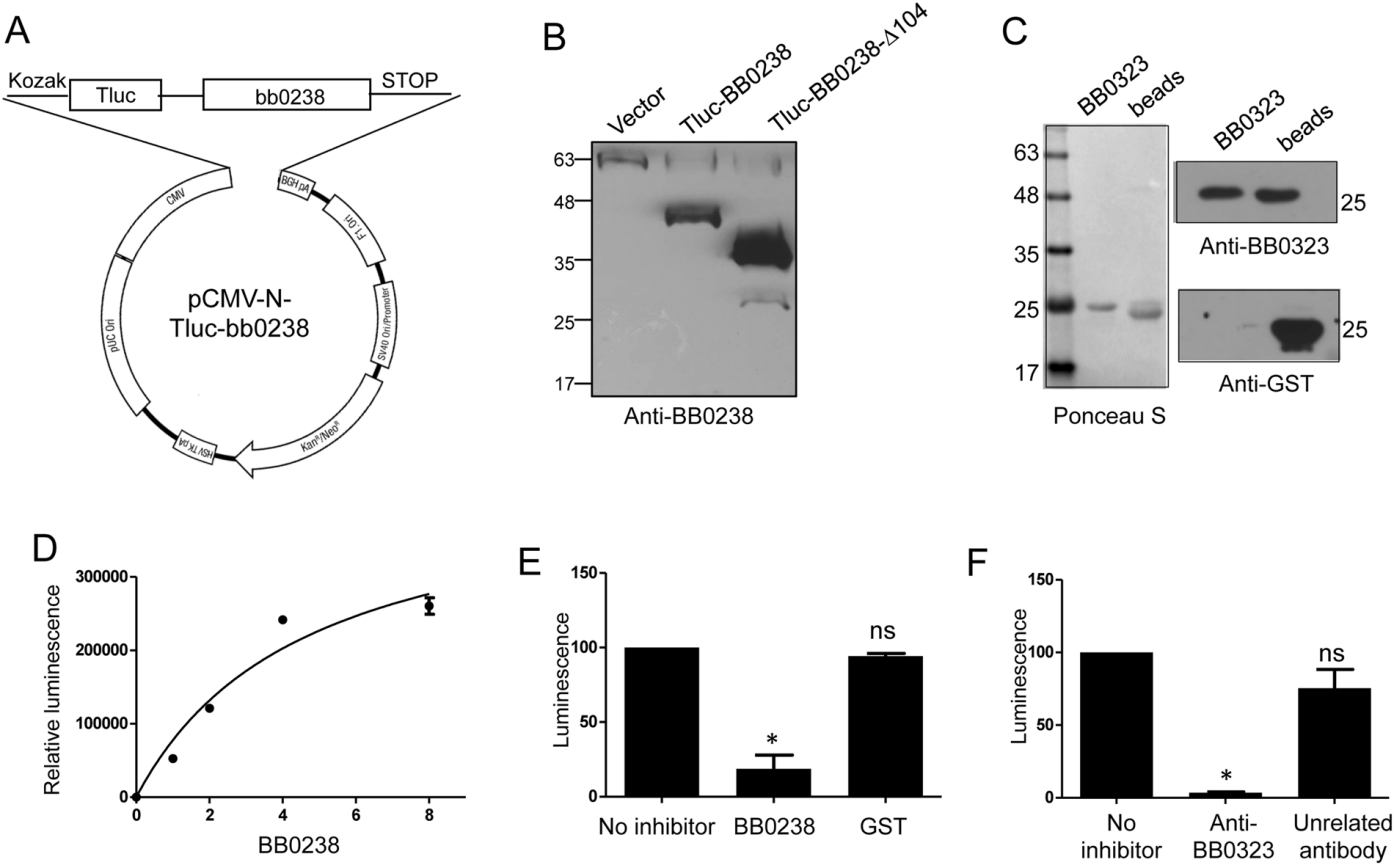
Development of a high throughput screening- compatible and luciferase based assay to monitor BB0238-BB0323 interaction. (**A**) Vector map of pCMV N-Tluc-bb0238 for the expression of BB0238 fused to TurboLuc (Tluc) Luciferase. The cytomegalovirus (CMV) promoter allows strong expression of TurboLuc-BB0238. (**B**) Production of BB0238 or derivatives in transfected mammalian cells. Immunoblot analyses of 293 T cells transfected either with empty vector (lane 1), or luciferase fused BB0238 (full length or truncated BB0238-∆104, lanes 2 and 3) probed with BB0238 specific antisera. (**C**) Purification of BB0323. BB0323-N-terminus (residues 22–225) was cleaved from GST using PreScission protease and the supernatant fraction carrying purified BB0323 and post cleavage beads were probed with anti-BB0323 and anti-GST antibody to rule out GST contamination in BB0323. (**D**) BB0238 binding to BB0323 measured by a luciferase assay. A 96-well white opaque plate was coated with BB0323 (0.1 µg/well), blocked with 5% BSA and incubated with increasing amount of lysates expressing BB0238 in fusion with luciferase (Tluc-BB0238). After washing, bound proteins were detected using luciferase substrate solution and the relative luminescence unit was measured using a luminometer. Binding was calculated after subtracting the blank (wells incubated with increasing amount of extracts transformed with empty vector) mean ± standard error of the mean. (E and F). Competition of BB0238-BB0323 binding. The binding of Tluc-BB0238 with immobilized BB0323 was determined in the presence of recombinant BB0238 purified from *E*. *coli* (*p < 0.05, ns - non significant) (**E**) or antibody specific to BB0323 (*p < 0.01). (**F**). The luciferase activity in the absence of any competitor was considered as 100%, and the results are expressed as the percentage of Tluc-BB0238 bound to BB0323. One- or two-tailed Student’s t tests were used to compare the mean values using GraphPad Prism.

As BB0238-BB0323 interaction is essential for infection of *B*. *burgdorferi*, we sought to develop a protein-protein assay that could be use to screen inhibitors or potentially small molecules that inhibit this interaction as a strategy to combat *B*. *burgdorferi* infection. We focused on luciferase assays that are sensitive, rapid, and adoptable to future high throughput screening format. To explore this, we expressed BB0238 as a fusion protein with luciferase in 293 T cells (Fig. 6A and B), and monitored the fusion protein binding with the N-terminal domain of BB0323 (Fig. 6C). The results show that binding of BB0238 to BB0323 is saturable (Fig. 6D) and that both recombinant BB0238 (Fig. 6E) and anti-BB0323 antisera (Fig. 6F) effectively inhibited the binding of BB0238 to BB0323, further confirming the specificity and validity of the assay for screening potential inhibitors that can block BB0238-BB0323 interaction.

## Discussion

Protein-protein interactions that facilitate many critical cellular processes and influence the biological functions constitute promising target for small-molecule therapeutics^23^. The Lyme disease agent *B*. *burgdorferi* features a unique genome with hundreds of proteins of unknown function, contributing in the special biology and infectivity of the spirochetes^15, 24^. Some of these novel proteins are likely to be involved is protein-protein interactions. In fact, the outer membrane (OM) of spirochetes harbor many protein complexes although their biological significance remains undefined^12^. Identifying novel protein-protein interactions and defining their roles in *B*. *burgdorferi* infectivity could shed new light on spirochete biology and virulence. The discovery of novel protein complexes required for spirochete infectivity could also contribute to the identification of new therapeutic approaches to combat the infection, including development of compounds that selectively target and inhibit a specific protein-protein interaction^23^. We have now characterized a recently identified protein-protein interaction involving two key virulence determinants in *B*. *burgdorferi*, BB0323 and BB0238, both of which are critical for infectivity^16, 19, 22^. Our current study not only highlighted the importance of this protein-protein interaction in the persistence and transmission of Lyme disease pathogens but also discovered that the BB0238 binding epitope comprises a relatively small 11-residue region, which could potentially be targeted by new antimicrobials that block infection.

Although BB0323 and BB0238 have recently been identified as borrelial virulence determinants^16, 19, 22^, both proteins are unrelated to known proteins and therefore, their precise functions in spirochete biology and infectivity remain enigmatic. BB0323 contains a putative LysM domain, whose function has not yet been determined. LysM domains, typically present in bacterial autolysins involved in cell division, recognize polysaccharides containing *N*-acetylglucosamine residues including peptidoglycan (PG)^19, 25^. The BB0323 LysM binds borrelial PG, however, deletion of the LysM motif did not exert any effect on spirochete cell fission or OM organization^17^. For BB0238, point mutations in the region spanning residues 34–67 influence protein stability or spirochete infectivity, and it was suggested that this region comprises a TPR motif, which could be involved in protein-protein interaction^22^. However, our sequence analysis and interrogation of databases does not support this notion and the role of the 34–67 region in protein-protein interaction has not been empirically demonstrated. Our study clearly shows that BB0323 and BB0238 interaction is independent of the 34–67 region, therefore, perhaps this region is involved in other unknown aspects of *B*. *burgdorferi* biology facilitating spirochete infection in mammals.

Secondary structure prediction suggests that residues 120–130 housing the BB0323 interaction motif in BB0238 form a α-helix, but the same region also has propensity to be intrinsically disordered. Protein-protein interactions are usually mediated by surface residues, and eight out of the eleven amino acids deleted in BB0238^*∆IM*^ have charged or polar side chains characteristic of protein surfaces. None of the single point mutations (to alanine) was sufficient to abolish the interaction between the two proteins, however the interaction was eliminated by replacing all 11 amino acids with alanine residues, and it was severely impaired upon replacement of the first 5 amino acids (KIEYI) for alanines (Supplementary Fig. S1). We speculate that a combination of a subset of surface-exposed residues within this region contributes to both self oligomerization and the association with BB0323. BB0323 undergoes post-translational maturation by at least two *B*. *burgdorferi* proteases^17, 26^. These cleavages ultimately yield two BB0323 polypeptides, which have distinct roles in spirochete biology and infectivity^17^. Whether or how BB0238 influences the maturation of the BB0323 polypeptides remains unknown. The fact that deletion or alanine substitution of the 11-residue interaction region in BB0238 did not alter the cellular abundance of the protein yet it affected levels of BB0323 polypeptides, either by impairing BB0323 maturation events or stability of the polypeptides. The function of BB0238 could depend on its optimal oligomerization property, which seems to be affected by the deletion or alteration of the 11-residue BB0323 interaction region.

BB0323 and BB0238 are members of two protein families that are conserved in diverse strains of Lyme disease agents prevalent globally and are consistently expressed throughout the infection cycle^16, 19^. As their interaction is essential for pathogen persistence or transmission, future studies to identify inhibitors could lead to novel therapeutics for *B*. *burgdorferi* infection. Drug development that targets protein-protein interactions that are essential to the survival or infectivity of persistent pathogen, such as *Mycobacterium tuberculosis* ^8^ offers a relatively new strategy that has not been much exploited. This strategy is especially relevant for Lyme borreliosis, which display perplexing and antibiotic-refractory episodes of chronic Lyme disease or post-treatment Lyme disease syndrome following conventional antimicrobial therapy.

## Methods

### B. burgdorferi, Mice, and Ticks

The *B*. *burgdorferi* infectious isolate B31-A3^27^ used in this study was grown in Barbour-Stoenner-Kelly-H (BSK-H) medium containing 6% rabbit serum at 33 °C. Generation of *bb0238* deletion mutant and *bb0238* complement were described elsewhere^16^. The *I*. *scapularis* ticks were reared in the lab as described elsewhere^28^. Four-to-six weeks old C3H/HeN mice were purchased from National Institutes of Health. All animal experiments were performed in accordance with the guidelines of the Institutional Animal Care and Use Committee and Institutional Biosafety Committee of the University of Maryland, College Park, who approved all experimental protocols used in the current manuscript.

### Western Blotting and far-Western Blotting

Immunoblotting was performed as detailed^28^, using antisera directed against BB0323, BB0238, FlaB or GST^16^. Immune complexes were detected by enhanced chemiluminescence. For far-Western analysis^17^, recombinant proteins were fractionated on 12% SDS-PAGE, and transferred to nitrocellulose membrane, which was blocked with 3% skim milk in TBST, incubated either with BB0323 or GST-fused proteins in blocking buffer overnight at 4 °C. After washing proteins were detected using anti-BB0323 or anti-GST antibody and HRP-conjugated anti-goat secondary antibody.

### Yeast two-hybrid assay

Yeast two-hybrid assays were performed as detailed^16^ using strain Y2H gold (MATa, trp1-901, leu2-3, 112, ura3-52, his3-200, gal4Δ, gal80Δ, LYS2:: GAL1UAS–Gal1TATA–His3, GAL2UAS–Gal2TATA–Ade2 URA3:: MEL1UAS–Mel1TATA AUR1-C MEL1). Plasmids encoding fusion proteins between GAL4 DNA BD-BB0323 (pGBKT7-BB0323) or the GAL4 AD-BB0238 (pGADT7-BB0238) were constructed earlier^16^. To assess interactions between BB0323 and BB0238, pGBKT7 or the pGBKT7 derivative expressing BB0323 was cotransformed into strain Y2H gold with pGADT7 or the pGADT7 derivatives expressing full length or truncated BB0238 fusion proteins. The interaction between BB0238-BB0323 was measured by blue colonies on plates containing X-α-galactosidase and confirmed either by stimulation of the ADE and HIS3 reporter in Y2H gold as assayed by growth on medium lacking Ade and His or resistance to Aureobasidin A, a cyclic depsipeptide antibiotic which is toxic to yeast at low concentration.

### Protein Turnover Assay

Stability of BB0323 was analyzed by a turnover assay, as described previously^16^. Briefly, wild type, *bb0238* mutant, *bb0238* complement and *bb0238* ^*∆IM*^ complement cultures were incubated in BSK-H medium containing spectinomycin (100 µg/mL) at 33 °C for 24 hours. Spirochetes were removed after 3, 6, and 24 hours and processed for immunoblotting using anti-BB0323 and anti-FlaB antibody.

### PCR and site-directed mutagenesis

The primers used in PCR and quantitative reverse-transcription PCR (qRT-PCR) are listed in Supplementary Table 1. For qRT-PCR, RNA was isolated using TRIzol reagent (Invitrogen), treated with DNaseI (NEB), reverse transcribed to complementary cDNA (Superscript VILO, Invitrogen), and analyzed by qRT-PCR using SYBR Green (Life Technologies) as described elsewhere^28^. For quantitative analysis of gene expression, *bb0323* transcripts were normalized to the number of *flaB* transcripts. Deletion and point mutations of *bb0323* and *bb0238* were generated either by overlap extension PCR or using Q5 site directed mutagenesis kit (NEB).

### Production and purification of recombinant proteins

BB0238, BB0238^*∆IM*^ and BB0323 (residues 22–242) proteins, partially purified using Glutathione S-transferase (GST) affinity column, contained DNA contaminant, detectable by high absorbance in 240–260 nm range. To obtain an accurate protein concentration, the GST column elutes were further purified using the Sephacryl S100 size exclusion chromatography (GE Lifesciences), equilibrated in 50 mM Tris-HCl (pH 8.0), 0.15 M NaCl, 0.5 mM ethylene-diamine-tetra-acetic acid (EDTA), 0.02% (w/v) NaAzide. The size exclusion chromatography was conducted at 0.5 ml/min flowrate and 1 mL fractions were collected. Prior to pooling, the eluted fractions were analyzed for protein purity with denaturing SDS-PAGE. The main contaminants eluted close to the column void volume. Protein concentrations were determined using the calculated extinction coefficient of 24,410 M^−1^ cm^−1^ for BB0238, 22,920 M^−1^ cm^−1^ for BB0238^*∆IM*^ and 27,390 M^−1^ cm^−1^ for BB0323 at 280 nm.

### Analytical Size Exclusion Chromatography

Analytical size exclusion chromatography was used to analyze the quaternary structure of BB0238 and BB0238^*∆IM*^ and BB0323 proteins. Blue dextran (2 × 10^6^), Horse spleen ferritin (440,000), Bovine serum albumin (68,000), Hen egg Ovalbumin (43,000), Chymotrysinogen A (25,600) and Bovine pancreas RNase A (13,700) were used to calibrate the Superdex 200 HR (10/300) and Superdex 75 (10/300) column in above buffer at 4 °C. The standard curves, a plot of the apparent molecular mass of protein standards as a function of their elution coefficients, were used to estimate the apparent molecular mass of proteins.

Secondary structure predictions of BB0238 and BB0323 were performed using the Robetta and I-tasser on line servers at http://robetta.bakerlab.org and http://zhanglab.ccmb.med.umich.edu/I-TASSER/.

### Dimethyl-suberimidate crosslinking

The proteins were treated with the protein crosslinking reagent, dimethyl-suberimidate. HCl (DMS) (Pierce), which reacts with lysine residues (Hartman & Wold, 1967, Davies & Stark, 1970). DMS was dissolved in 1 M Triethanolamine, pH 8.5, immediately before use. BB0238 WT or BB0238^∆IM^ (10–15 μM) in 20 μL reaction volume was mixed with 1 μL of 25 μg/μL DMS adding aliquots every 15 minutes to a final concentration of 5 μg/μL. After 1 hour incubation at 22 °C, the reaction products were separated by denaturing SDS-PAGE and visualized by Coomassie Blue (CB) staining.

### Genetic manipulation of *B*. *burgdorferi*

Genetic complementation of the *bb0238* mutant with *bb0238* ^*∆IM*^ was achieved by insertion of a copy of *bb0238* devoid of 11 amino acid residues in the chromosome of *B*. *burgdorferi*. To accomplish this, the original complementation plasmid pXLF14301-*bb0238* ^16^ was used as template to generate pXLF14301-*bb0238* ^*∆IM*^ using the Q5 mutagenesis kit (NEB). The final construct was sequenced to confirm the deletion and electroporated into the *bb0238* mutant *B*. *burgdorferi*. One of the *bb0238* ^*∆IM*^ complemented clones that grew on a double antibiotic selection (kanamycin and streptomycin) was tested for the intended recombination event by sequencing of the amplified product and monitoring the expression of BB0238^∆IM^ protein by immunoblotting. The complemented strain was verified for the presence of all the plasmids and used for subsequent analysis (Supplementary Fig. S4). To further confirm that the observed effects were not an outcome of undesirable genomic alteration, *bb0238* was also complemented at the native locus with *bb0238* ^*∆IM*^. Briefly, the complementation construct pXLF10301-*bb0238*com generated previously was subjected to deletion mutagenesis to generate pXLF10301- *bb0238* ^*∆IM*^com. Two clones from independent transformation harboring entire set of essential plasmids were tested for the expression of protein and analyzed for infection in murine hosts.

### Infection studies using mice and ticks

To assess spirochete persistence in rodent hosts, groups of C3H/HeN mice (3 animals/group) were injected intradermally with equal number (10^5^ cells/per mouse) of *bb0238* deletion mutant, *bb0238*-complemented or *bb0238* ^*∆IM*^-complemented spirochetes, as detailed^16^. Sera collected two weeks post infection was used to assess immunological response against whole cell extracts from *B*. *burgdorferi*. Samples of skin, heart, and bladder were isolated at 14 days after infection, and *B*. *burgdorferi* level was assessed using qRT-PCR analysis of *flaB* transcripts normalized against murine *β-actin* levels, as described^16^.

For studies involving pathogen transmission from ticks to mice, nymphs were microinjected with 10^9^ wild type (WT), *bb0238* deletion mutant (*bb0238* ^−^), *bb0238* complement (*bb0238*com) and *bb0238* ^*∆IM*^ complement (*bb0238* ^*∆IM*^) isolates of *B*. *burgdorferi*, as detailed^28^. The infected ticks were fed on naïve mice (5 ticks per mouse, 3 mice per group) and fully engorged ticks were subjected to qRT-PCR analyses. Mice were euthanized 10 days following tick feeding, tissues were isolated and assessed for spirochete burden by qRT-PCR.

### Luciferase based BB0238-BB0323 interaction assay

The open reading frame corresponding to *bb0238* was cloned in the pCMV-N-Tluc vector (Thermo Scientific) using *B*. *burgdorferi* genomic DNA as template. The fusion construct along with the empty vector for use as control was transfected in 293 T cells and the extracts were prepared 40 h post transfection. The extracts were verified for the expression of BB0238 by immunoblotting using anti-BB0238 antiserum. The N-terminal domain (residues 22–225) of the binding partner BB0323 was purified using Glutathione Sepharose 4B resin following the manufacturer’s recommended protocol and the cleaved protein was utilized for the luciferase interaction assay.

For the interaction assay, 100 µL of BB0323 (1 µg/mL) was coated on white opaque flat-bottom 96-well plates (Thermo Scientific) in PBS at 4 °C overnight. The plate was blocked with 5% BSA for 1 h at room temperature. Increasing amount of whole cell extracts from 293 T cells expressing BB0238 in fusion with luciferase or vector control, were added to the BB0323 microtiter plate wells. After thorough washing, 50 μL of luciferase substrate solution (1:100 diluted, Thermo Scientific) was added to each well, incubate for 10 minutes and the relative light units (RLU) were measured in a luminometer (TECAN Infinite M1000).

## Electronic supplementary material

Supplementary information
