# Supplementary material for: A protein-protein interaction dictates Borrelial infectivity
Source: Sci Rep. 2017 Jun 7;7:2932. doi: 10.1038/s41598-017-03279-7 (PMC5462797; doi:10.1038/s41598-017-03279-7)
Supplement: Supplementary file 1 — Supplementary information [file 41598_2017_3279_MOESM1_ESM.pdf]

Supplementary Information for the manuscript entitled

"A protein-protein interaction dictates Borrelial infectivity"

Meghna Thakur, Kavita Sharma, Kinlin Chao, Alexis A. Smith,
Osnat Herzberg and Utpal Pal

The supplementary information contains:

Four Supplementary Figures
Legends to Supplementary Figures
Supplementary Table

Figure S1

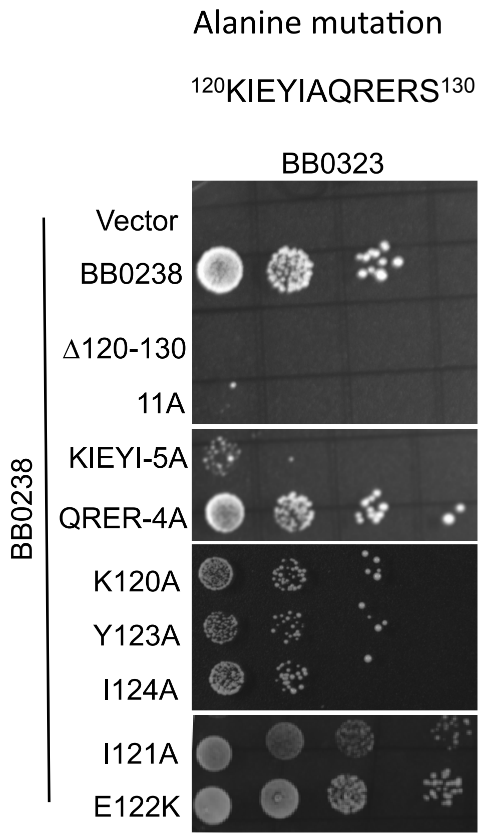

Alanine mutation
^{120}KIEYIAQRERS^{130}
BB0323
Vector
BB0238
Δ120-130
11A
KIEYI-5A
QRER-4A
K120A
Y123A
I124A
I121A
E122K
BB0238

Figure S2

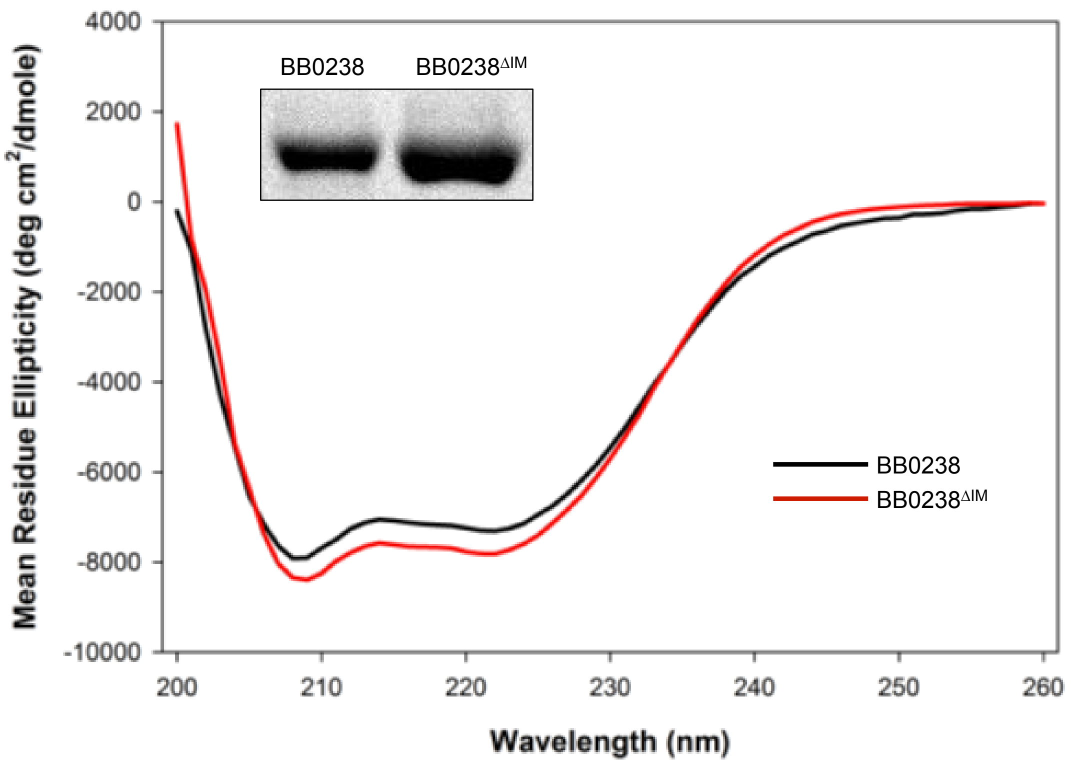
BB0238
BB0238ΔIM
4000
2000
0
-2000
-4000
-6000
-8000
-10000
Mean Residue Ellipticity (deg cm^2/dmole)
200
210
220
230
240
250
260
Wavelength (nm)
BB0238
BB0238ΔIM

Figure S3

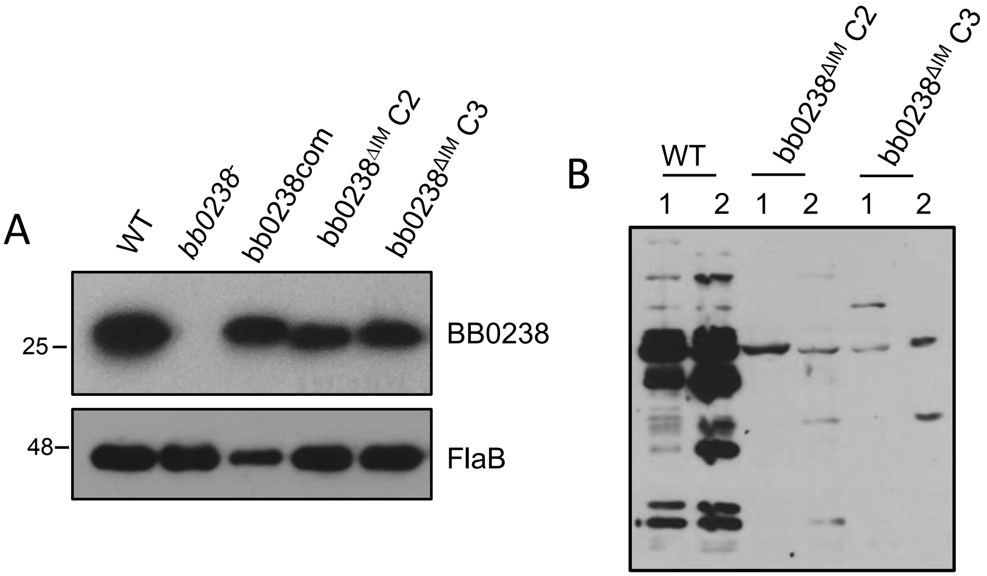
A
WT
bb0238-
bb0238com
bb0238ΔIM C2
bb0238ΔIM C3
25
48
BB0238
FlaB
B
WT
bb0238ΔIM C2
bb0238ΔIM C3
1 2 1 2 1 2

Figure S4

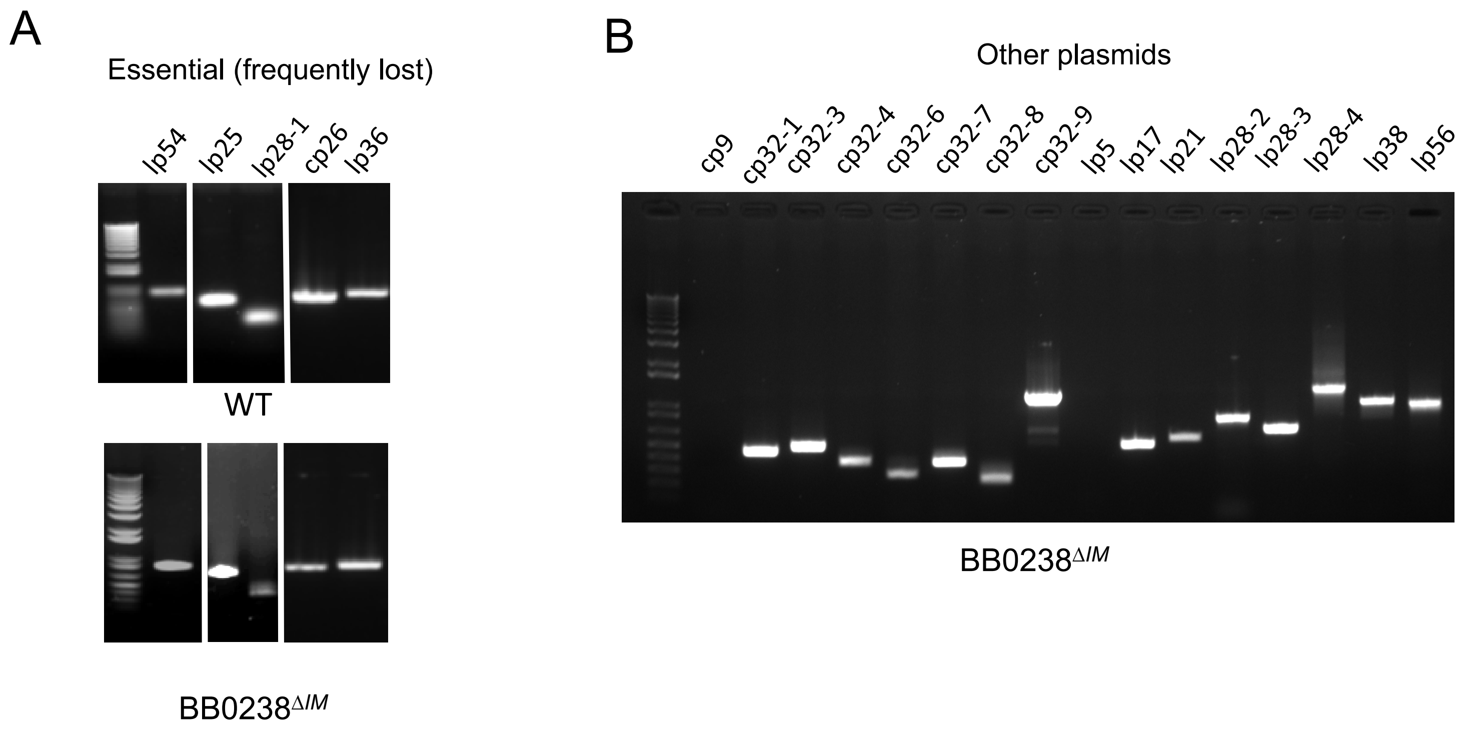
A
Essential (frequently lost)
lp54
lp25
lp28-1
cp26
lp36
WT
BB0238$^{\Delta IM}$
B
Other plasmids
cp9
cp32-1
cp32-3
cp32-4
cp32-6
cp32-7
cp32-8
cp32-9
lp5
lp17
lp21
lp28-2
lp28-3
lp28-4
lp38
lp56
BB0238$^{\Delta IM}$

Figure S5 (Related to figure 1B)

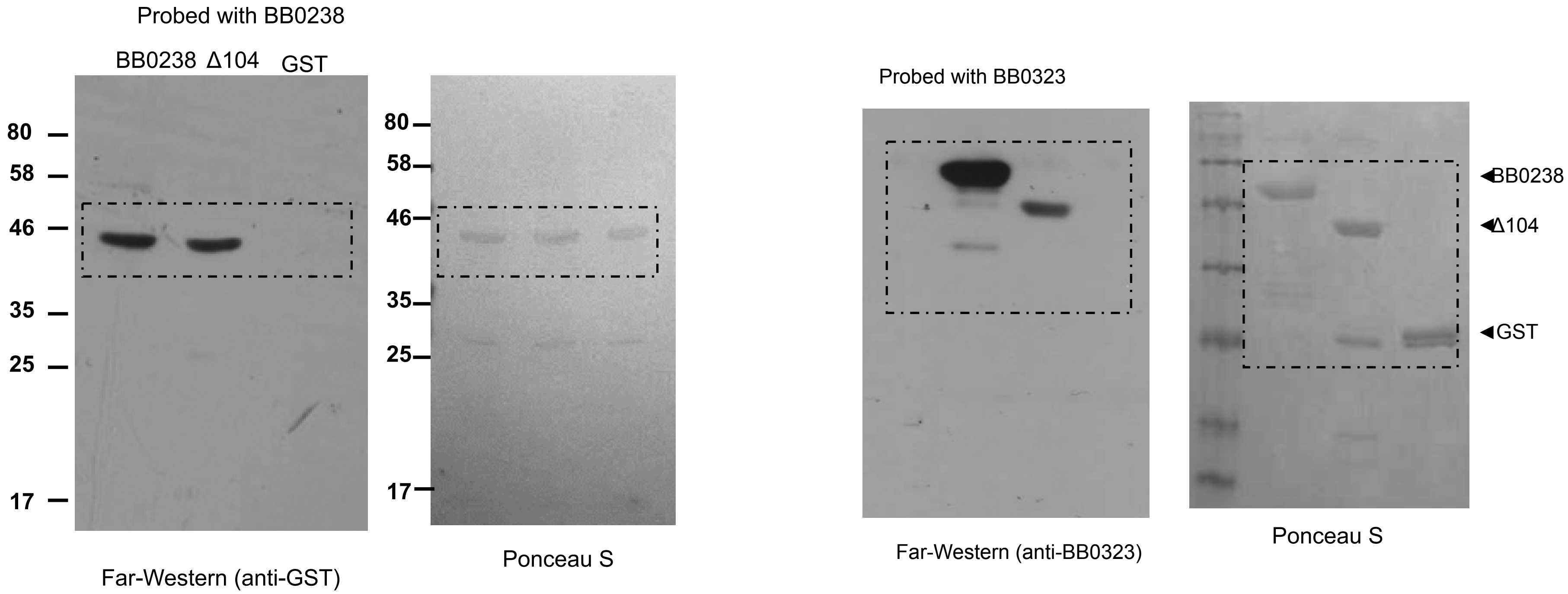

Figure S6 (Related to figure 1D)

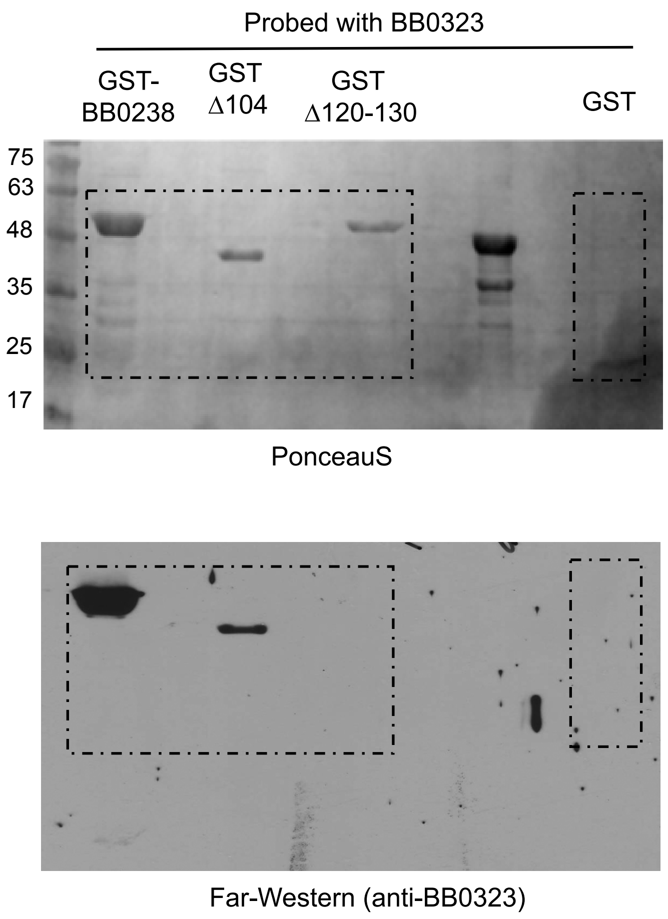

Legends to Supplementary Figures

Figure S1. Alanine scanning mutagenesis of the interaction motif of BB0238. Mutation of a combination (KIEYI or QRER) or individual (K120A. I121A, E122K, Y123A and I124A) amino acid residues of BB0238 encompassing the interaction motif were assessed for interaction with BB0323 in yeast two hybrid.

Figure S2. BB0238$^{\Delta IM}$ is not a misfolded protein. Far-ultraviolet (UV) circular dichroism (CD) spectra of BB0238 and BB0238$^{\Delta IM}$ proteins. CD spectra from 200 to 260 nm for BB0238 (black line) and BB0238$^{\Delta IM}$ (red line) are presented as mean residue ellipticity (deg-cm2/dmol) versus wavelength (nm) after baseline subtraction of the buffer. The spectra indicated that both wild type and mutant proteins retained similar secondary structure content, predominantly alpha helix. Inset shows purified proteins used for CD.

Figure S3. Complementation of bb0238 mutant *B. burgdorferi* with *bb0238*$^{\Delta IM}$ at the native locus does not restore the infectivity of spirochetes in mice. (A) Immunoblot showing the restoration of expression of BB0238 in the two independent complemented isolates (*bb0238*$^{\Delta IM}$ C2 and *bb0238*$^{\Delta IM}$ C3). (B) Immunoblot showing the serological profile in mice of complemented clones along with the WT.

Figure S4. Endogenous plasmid profile of bb0238ΔIM. bb0238ΔIM isolate contains same set of plasmids as wild-type (WT) except for a non-essential plasmid lp5. DNA was isolated from *B. burgdorferi* isolates and used in polymerase chain reaction for detection of endogenous plasmids using specific primer sets.

Figure S5. (Related to Figure 1B) Uncropped Western blot of BB0323 and BB0238 interaction in Far-Western assays. Recombinant BB0323 (left panels) or BB0238 (right panels) was subjected to SDS-PAGE and incubated with glutathione S-transferase (GST)-fused BB0238, or

His-tagged BB0323 proteins, and binding was examined by appropriate primary and secondary detection antibodies.

Figure S6. (Related to Figure 1D) Uncropped Western blot far Western assay. BB0238 missing 11-residue interaction motif failed to bind BB0323 in far-Western assays. Recombinant GST-BB0238 fusion proteins were subjected to SDS-PAGE and incubated with recombinant his-tagged BB0323 and the interaction was monitored as described in panel

Supplementary Table S1. Oligonucleotide primers used in the current study

Sequence (5’→3’)	Purpose
TTGCTGATCAAGCTCAATATAACCA	Forward primer for *B. burgdorferi flaB* qRT-PCR
TTGAGACCCTGAAAGTGATGC	Reverse primer for *B. burgdorferi flaB* qRT-PCR
AGAGGGAAATCGTGCGTGAC	Forward primer for mouse *β-actin* qRT-PCR
CAATAGTGATGACCTGGCCGT	Reverse primer for mouse *β-actin* qRT-PCR
AGAGGGAAATCGTGCGTGAC	Forward primer for tick *β-actin* qRT-PCR
CAATAGTGATGACCTGGCCGT	Reverse primer for tick *β-actin* qRT-PCR
ATATGGATCCCGCTGGAAAT	Forward primer for RT-PCR of *bb0323*
AGCCGCTTCAAGTGCTTTTA	Reverse primer for RT-PCR of *bb0323*
AAT*GAATTC*GTTGGTAAAATTAAAAAAGGAAAGC	Forward primer for cloning of bb0238-Δ46 in pGADT7 plasmid. An EcoRI site (italicized) is attached for the purpose of cloning
AAT*GAATTC*ATAGTGCACGAAAAAATAGAAATC	Forward primer for cloning of bb0238-Δ104in pGADT7 plasmid. An EcoRI site (italicized) is attached for the purpose of cloning
AAT*GAATTC*GTTATGGGTCAATTTGAATCAAAAAATG	Forward primer for cloning of bb0238-Δ165 in pGADT7 plasmid. An EcoRI site (italicized) is attached for the purpose of cloning
AAT*GAATTC*TTTACAAATGCTCCTAAAATAGAA	Forward primer for cloning of bb0238-Δ115 in pGADT7 plasmid. An EcoRI site (italicized) is attached for the purpose of cloning
AAT*GAATTC*AGCAAAAATCAAGATAAAATTATTAAG	Forward primer for cloning of bb0238-Δ130 in pGADT7 plasmid. An EcoRI site (italicized) is attached for the purpose of cloning
AAT*GAATTC*TCTAGGAACTTTGATTTGTTTGATTC	Forward primer for cloning of bb0238-Δ149 in pGADT7 plasmid. An EcoRI site (italicized) is attached for the purpose of cloning
AAAAATCAAGATAAAATTATTAAGTTTCAATTTG	Forward primer for deletion of bb0238-Δ120-130
AGGAGCATTTGTAAAATCATTG	Reverse primer for deletion of bb0238-Δ120-130
TGCAGCAGCGGCAGCCAAAAATCAAGATAAAATTATTAAGTTTCAATTTG	Forward primer for simultaneous substitution of bb0238-120-130 to alanine
GCAGCAGCTGCTGCTGCAGGAGCATTTGTAAAATCATTG	Reverse primer for simultaneous substitution of bb0238-120-130 to alanine
AGCTGCTGCTCAAAGAGAGAGAAGC	Forward primer for substitution of KIEYI to alanine
GCTGCTGCAGGAGCATTTGTAAAATCATTG	Reverse primer for substitution of KIEYI to alanine
GCGGCAAGCAAAAATCAAGCTAAAATTATTAAG	Forward primer for substitution of QRER to alanine
TGCTGCAGCAATATATTCTATTTTAGGAGC	Reverse primer for substitution of QRER to alanine
AAATGCTCCTGCAATAGAATATATTGCTC	Forward primer for K120A mutation
GTAAAATCATTGATTTCTATTTTTTCG	Reverse primer for K120A mutation
TGCTCCTAAAGCAGAATATATTGCTCAAAG	Forward primer for I121A mutation

TTTGTAAAATCATTGATTTCTATTTTTTC	Reverse primer for I121A mutation
TCCTAAAATAAAATATATTGCTCAAAG	Forward primer for E122K mutation
GCATTTGTAAAATCATTGATTTCTATTTTTTC	Reverse primer for E122K mutation
TAAAATAGAAGCTATTGCTCAAAGAGAGAGAAG	Forward primer for Y123A mutation
GGAGCATTTGTAAAATCATTG	Reverse primer for Y123A mutation
AATAGAATATGCTGCTCAAAGAGAGAGAAG	Forward primer for I124A mutation
TTAGGAGCATTTGTAAAATCATTG	Reverse primer for I124A mutation
GCGGAATTCAAAACGCCTCCAGAATCAAGA	Forward primer for BB0323 (22-225) in pGBKT7
AATCTGCAGTTATCTACCATTCCATGGAGATGG	Reverse primer for BB0323 (22-225) in pGBKT7
AAT*CTGCAG*TTATTTTAATTGTTTGTACATTCTCTC	Reverse primer for BB0323 (22-200) in pGBKT7
AATCTGCAGTTAATCGTAATTTTTATATTTTCTTGT	Reverse primer for BB0323 (22-160) in pGBKT7
GCGGAATTCAGATACAAAACAATAATAAGAGAA	Forward primer for BB0323 (100-225) in pGBKT7
GCGGAATTCGGACATCTTTTTTATTCCAAAGAG	Forward primer for BB0323 (60-225) in pGBKT7
GCGGAATTCAGAGCATTCATTAAAGAAAGAAAC	Forward primer for BB0323 (225-377) in pGBKT7
AATCTGCAGTTATTTGGCAGGAATTATTAT	Reverse primer for BB0323 (225-377) in pGBKT7
